# Host-pathogen interplay at primary infection sites in pigs challenged with *Actinobacillus pleuropneumoniae*

**DOI:** 10.1186/s12917-017-0979-6

**Published:** 2017-02-28

**Authors:** Elena L. Sassu, Janna Frömbling, J. Catharina Duvigneau, Ingrid Miller, Andrea Müllebner, Ana M. Gutiérrez, Tom Grunert, Martina Patzl, Armin Saalmüller, Alexandra von Altrock, Anne Menzel, Martin Ganter, Joachim Spergser, Marion Hewicker-Trautwein, Jutta Verspohl, Monika Ehling-Schulz, Isabel Hennig-Pauka

**Affiliations:** 1 University Clinic for Swine, Department of Farm Animals and Veterinary Public Health, University of Veterinary Medicine Vienna, Vienna, Austria; 2 Department of Pathobiology, Functional Microbiology, Institute of Microbiology, University of Veterinary Medicine Vienna, Vienna, Austria; 3 Department of Biomedical Sciences, Institute for Medical Biochemistry, University of Veterinary Medicine Vienna, Vienna, Austria; 4 Department of Animal Medicine and Surgery, University of Murcia, Murcia, Spain; 5 Department of Pathobiology, Institute of Immunology, University of Veterinary Medicine Vienna, Vienna, Austria; 6 Forensic Medicine and Ambulatory Services, Clinic for Swine and Small Ruminants, University of Veterinary Medicine Hannover, Hannover, Germany; 7 Department of Pathology, University of Veterinary Medicine Hannover, Hannover, Germany; 8 Institute for Microbiology, University of Veterinary Medicine Hannover, Hannover, Germany

**Keywords:** FTIR, Salivary gland, Acute phase proteins, Early immune response, Gene expression

## Abstract

**Background:**

*Actinobacillus (A.) pleuropneumoniae* is the causative agent of porcine pleuropneumonia and causes significant losses in the pig industry worldwide. Early host immune response is crucial for further progression of the disease. *A. pleuropneumoniae* is either rapidly eliminated by the immune system or switches to a long-term persistent form. To gain insight into the host-pathogen interaction during the early stages of infection, pigs were inoculated intratracheally with *A. pleuropneumoniae* serotype 2 and humanely euthanized eight hours after infection. Gene expression studies of inflammatory cytokines and the acute phase proteins haptoglobin, serum amyloid A and C-reactive protein were carried out by RT-qPCR from the lung, liver, tonsils and salivary gland. In addition, the concentration of cytokines and acute phase proteins were measured by quantitative immunoassays in bronchoalveolar lavage fluid, serum and saliva. In parallel to the analyses of host response, the impact of the host on the bacterial pathogen was assessed on a metabolic level. For the latter, Fourier-Transform Infrared (FTIR-) spectroscopy was employed.

**Results:**

Significant cytokine and acute phase protein gene expression was detected in the lung and the salivary gland however this was not observed in the tonsils. In parallel to the analyses of host response, the impact of the host on the bacterial pathogen was assessed on a metabolic level. For the latter investigations, Fourier-Transform Infrared (FTIR-) spectroscopy was employed. The bacteria isolated from the upper and lower respiratory tract showed distinct IR spectral patterns reflecting the organ-specific acute phase response of the host.

**Conclusions:**

In summary, this study implies a metabolic adaptation of *A. pleuropneumoniae* to the porcine upper respiratory tract already during early infection, which might indicate a first step towards the persistence of *A. pleuropneumoniae*. Not only in lung, but also in the salivary gland an increased inflammatory gene expression was detectable during the acute stage of infection.

**Electronic supplementary material:**

The online version of this article (doi:10.1186/s12917-017-0979-6) contains supplementary material, which is available to authorized users.

## Background

*Actinobacillus (A*.) *pleuropneumoniae* is the etiological agent of porcine contagious pleuropneumonia, which results in increased mortality throughout swine production worldwide [1]. The outcome of infection ranges from colonisation of the upper respiratory tract without any clinical signs to severe lung infection with peracute death. The severity has been ascribed to variation in serotype-related virulence, influenced by biotic and abiotic factors in the pig environment [1]. If the pig overcomes the acute phase of the disease, it can harbour the bacterium in chronic lung lesions, tonsillar crypts and nasal cavities. Thus, infected pigs become persistent carriers of the infectious agent [2]. The Waldeyer’s ring is the first immunological and mechanical barrier faced by inhaled pathogens [3, 4]. Its failure allows persistence of *A. pleuropneumoniae* at this site or may lead to reoccurrence of acute outbreaks. Early innate immune response to respiratory disease is not restricted to the lung as the primary site of infection, but additionally involves peripheral lymphoid tissues, the liver [5] and the salivary gland [6]. The acute immune response is characterised by the self-sustaining production of acute phase proteins and inflammatory cytokines. Particularly for *A. pleuropneumoniae* the synergic action of endotoxins and the pore forming exotoxins Apx I to IV, in enhancing the production of inflammatory cytokines, such as IL-6, TNF-α and IL-1 is well known [5, 7–9]. Thus, these bacterial virulence factors can cause tissue damage, directly by Apx cytotoxic effect and indirectly by mounting an exacerbated inflammatory response.

To gain insight into the early stages of host-pathogen interaction, we experimentally challenged pigs with *A. pleuropneumoniae* using the endotracheal infection route and studied in parallel the host and the pathogen during the first few hours of infection. We investigated the mRNA expression of inflammatory cytokines and acute phase proteins in the lung, liver, salivary gland and tonsils as well as the protein levels of these markers in bronchoalveolar lavage fluid (BALF), serum and saliva samples. For studying the influence of the host milieu on the bacterial pathogen, Fourier-Transform Infrared (FTIR) spectroscopy, was employed. FTIR spectroscopy is a well-established vibrational spectroscopic technique that can be used for the generation of spectral fingerprints from a broad range of biological materials [10, 11]. Recently, chemometric assisted FTIR spectroscopy was shown to be a valuable tool for studying metabolic adaptation of bacterial pathogens to host environments. For instance, FTIR spectroscopy was successfully applied for the examination of host genotype-specific imprints on the metabolism of *Listeria monocytogenes* re-isolated from mice with different genotypes [12]. In another study, FTIR spectroscopic analysis of *Streptococcus uberis* re-isolated during the progression of the uterine clearance process of post-partum cows revealed specific *S. uberis* biotypes, which could be linked to the uterine health status [13]. This renders FTIR spectroscopy a suitable technique to investigate bacterial host adaptation on a macromolecular and metabolic level not only for human but also for animal pathogens. In our current study, we showed that already in the early stages of porcine infection *A. pleuropneumoniae* undergoes organ specific metabolic changes that mirror those detected in the host.

## Methods

### Animals

Ten 6–8 week-old healthy pigs (German Landrace) were used in this study. Animals were derived from a closed breeding herd of a high health status that is routinely tested negative for *A. pleuropneumoniae*, Porcine Reproductive and Respiratory Syndrome Virus (PRRSV), toxigenic *Pasteurella multocida*, endo- and ectoparasites. After arrival, all pigs tested negative for *A. pleuropneumoniae* using an Apx-II Enzyme-linked immunosorbent assay (ELISA) [14]. At arrival, animals were individually marked with ear tags and randomly assigned to a control (*n* = 4) or infection (*n* = 6) group. Within a one-week adaptation period pigs became familiar to the housing conditions. Twice a day commercial feed was supplied. Pigs were housed under specific pathogen-free conditions according to FELASA guidelines and were continuously observed during the whole experiment. A humane intervention point (HIP) checklist, which has been developed and approved previously for infection experiments with *A. pleuroneumoniae*, was used during monitoring the animals continuously for clinical signs by trained staff (participants of FELASA B training course) [15, 16]. HIP was conducted by injection of 60 mg pentobarbital/kg body weight intravenously in deep anaesthesia of the animals as soon as behavioural changes or a significant increase in body temperature reflected the development of early respiratory disease.

### Experimental infection protocol

The experiment was part of a comprehensive study to characterise the early stages of inflammatory lung alterations by imaging techniques such as infrared thermography (data not published).

Pigs were not fed on the day of infection to decrease the risk of pulmonary aspiration of stomach content under anaesthesia. An acute infection trial lasting 8–10 h was carried out and included anaesthesia, surgical implantation of the central catheter into the *Vena cava cranialis*, intratracheal infection, clinical examination and collection of blood and saliva samples till scheduled euthanasia. Pigs were anaesthetized intramuscularly with 15 mg ketamine (Ursotamin®, Serumwerk-Bernburg AG, Bernburg, Germany) per kg body weight (bw) and 2 mg azaperon (Stresnil®, Janssen-Cilag GmbH, Baar, Switzerland) per kg bw. Intratracheal infection was performed under visible control using a flexible fiberoptic bronchoscope (IT3; Olympus, Hamburg, Germany) as previously described [15]. Briefly, the tip of the bronchoscope was placed cranial the *Bifurcatio tracheae* and 5 ml of inoculum was instilled gently into the main bronchi. *A. pleuropneumoniae* biotype 1-serotype 2 strain (no. C3656/0271/11, isolated from a fattening pig with respiratory symptoms during an acute outbreak of porcine pleuropneumonia in northern Germany and stored at the Institute of Microbiology, University of Veterinary Medicine, Hannover, Germany [17]) was cultivated in fresh pleuropneumonia-like organism (PPLO) liquid medium to reach an optical density of approximately 0.45 at 600 nm for infection as described elsewhere [18]. This culture was subsequently diluted 1:1000 with 154 mM sterile NaCl solution, resulting in a challenge dose of approximately 1.6x10^6^ CFU per pig determined retrospectively by serial dilution and overnight culture. Control pigs were treated with 154 mM sterile NaCl (sham control). Blood and saliva samples were taken at 2 h prior to and 2, 5 and 8 h post infection and clinical scores were recorded simultaneously.

Blood samples were collected by means of the central catheter into vacutainer tubes with either EDTA for haematological examination or sodium heparin for biochemical examination as anticoagulants.

Saliva samples were obtained by allowing pigs to chew a cotton wool swab (Salivette®, Numbrecht, Germany). The cotton wool swabs were replaced in the original tube, centrifuged at 3000 × g for 10 min and the supernatants stored at−20 °C. Rectal body temperature was measured every hour. At the end of the experiment all pigs were euthanized by intravenous application of 60 mg pentobarbital (Euthadorm®, CP-Pharma, Burgdorf, Germany)/kg bw at eight to ten hours after infection and necropsy was performed immediately.

### Clinical evaluation

Clinical examinations were carried out before infection and at 2, 5 and 8 h post infection (hpi) and a total clinical score was calculated for each animal at each time point. This total clinical score was obtained by summing up the single scores for six clinical parameters, as dyspnoea (heavy breathing = 1; open-mouth breathing = 2), coughing (=1), posture (sitting = 1; lying = 2), body temperature (38.0–39.5 °C = 0; >39.5 °C = 1; <38.0 °C = 2), vomiting (=1) and sudden death (=2 extra), so that an individual maximal clinical score of 10 was possible. Scores recorded at 8 hpi were statistically compared between the groups.

### Post-mortem examinations

After euthanasia lungs were removed immediately from the carcasses. The severity of lung alterations was assessed using the lung lesion score (LLS) proposed by Hannan et al. [19] and as recommended by the European Pharmacopoeia for the control of vaccine efficacy (3^rd^ edition, EDQM, Council of Europe, Strasbourg, France). Briefly, using a schematic map of the porcine lung as a guide, the organ was virtually subdivided into 74 triangles (7 triangles for cranial and middle lobes, 19 triangles for caudal lobes and 8 triangles for the accessory lobe). Then, the number of triangles with pathological lung alterations were expressed as a fraction and multiplied by five for each lobe, so that each lobe could reach a maximum score of 5, resulting in a maximum LLS of 35 when the entire lung was affected.

One main bronchus was separated from the lung by a surgical clamp. Lung tissue samples were taken from the clamped lung lobe, while the other lung lobe was lavaged with 100 ml of 154 mM sterile NaCl solution. Lung lavage fluid was collected by gently pouring in a glass container moistened inside with concentrated heparin solution. Tissue samples from the clamped main lung lobes were fixed in 10% formalin containing 2% calcium acetate for the preparation of paraffin sections. Routine histology sections were stained with hematoxylin-eosin (HE, hemalaun after Delafield). For mRNA extraction, approximately 500 mg tissue samples from liver, tonsils, salivary gland and lung were snap frozen in liquid nitrogen and then stored at −80 °C.

### Blood and lung lavage fluid analyses

Leucocyte cell counts and differential blood counts were determined (Haemotology analyser, Celltag alpha, Nihon Kohden, Kleinmachnow, Germany) immediately after blood collection. Serum haptoglobin (Hp) concentrations were analysed with a colorimetric method (Tridelta Phase Haptoglobin Assay, Tridelta Development Limited, Maynooth, Ireland), while serum C-reactive protein (CRP) was determined by ELISA (Phase Porcine CRP Assay, Tridelta Development Limited).

Total leukocyte counts were determined in the undiluted lavage fluid in a Neubauer-counting chamber prior to centrifugation of the fluid (10 min, 200 × g, 4 °C). The sediment was used for further cytological examination. Cytospots were prepared for differential cell determination of the bronchoalveolar lavage fluid (BALF) by centrifugation of small amounts of resuspended sediments in a cytocentrifuge (Multifuge KR®, Heraeus, Thermo, Osterode, Germany) at 200 × g for 10 min. Cells were stained with a Pappenheim staining solution (Merck, Darmstadt, Germany) and 400 cells were differentiated at 1000 × magnification. The cell-free supernatant of lung lavage fluid was stored at −80 °C.

### Analysis of gene expression in tissues

RNA was extracted and reverse transcribed as described previously [20]. The primer sequences and hybridization probes used for the detection of porcine cytokine mRNAs (IL-2, IL-4, IL-10, IL-6, INF-γ and IL-1) as well as the internal references (GAPDH, cyclophilin A and β-actin) were reported by Duvigneau et al. (2005) [21]. The primer sequences and hybridization probes for the detection of porcine stress gene mRNAs (iNOS/HO1/TNF-α) were detailed in a previous work [20]. The primer sequences used for the specific amplification of porcine CRP and Hp were described by Skovgaard et al. (2009) [5], and the primer sequence for amplification of porcine SAA was characterised by Soler et al. (2011a, b) [22, 23]. Primers used for the detection of IL-8 expression were designed for this study as follows: forward: AACAGCCCGTGTCAACATGA and reverse: TGCACTGGCATCGAAGTTCT. The suitability of the newly designed primers was verified in separate experiments by performing a dilution series using the PCR products as well as a dilution series of the cDNA pool, generated by collecting equal aliquots of all investigated cDNA samples. The dilution series, in conjunction with the melting characteristics of the PCR product, were used to optimise the assays regarding the primer concentration as well as the annealing and extension times for the PCR. Further details about the validation of the qPCR assays are provided (see Additional file 1). All primers (Invitrogen, Carlsbad, CA, USA) and probes (Eurofins MWG Operon, Ebersberg, Germany) were synthesised commercially. Specificity of the generated PCR products was verified using melt curve analysis and by randomly verifying correct fragment sizes using gel electrophoresis.

PCR assays were performed as described in [21, 24]. All samples were measured in duplicate. Each plate contained corresponding randomly assigned RT-minus controls of about 15% of all samples, the non-template controls (NTC) as well as the internal standard (IS), which was generated by pooling aliquots of all samples investigated in this study. Data were analysed as described previously [23] and normalised against cyclophilin A and β-actin. The obtained ∆∆Cq values of the replicates were averaged and expressed as fold change relative to the IS.

### Quantitative ELISA of serum and BALF

Serum and BALF samples were analysed for IL-6, TNF-α and IL-1 expression by means of commercially available sandwich ELISA assay (Duoset DY686, DY690B, DY681, R&D Systems, Biomedical medical products GmbH and Co KG, Vienna, Austria) according to the manufacturer’s instructions with minor modifications. For the preparation of standard curves, the recombinant cytokines were diluted in the same body fluid as the samples. Therefore, serum samples from four or BALF from two healthy age-matched pigs were pooled to decrease the risk of individual variation in the matrix. For serum samples, in order to increase signal intensity, the working concentrations of capture and detection antibodies and of the streptavidin-HRP conjugate were doubled. The respective recombinant protein was diluted serially (1:2) in the serum pool. Sera were analysed undiluted and all samples, controls and standard concentrations were run in duplicate. For BALF samples, the working concentrations of antibodies and HPR-conjugates were used as recommended. The BALF pool to create the standard curves was used in the same dilution as the samples. BALF was used undiluted for TNF-α and diluted 1:4 for IL-1 and IL-6 detection. The detection limits of the assays were 150 pg/ml for IL-6, 200 pg/ml for TNF-α and 70 pg/ml for IL-1, respectively. Tetramethylbenzidine (TMB) was used for colour development and 1 M sulphuric acid as stopping solution according to the manufacturer’s instructions. Optical density was measured at 450 nm and at 690 nm as the reference with an ELISA reader (Tecan, Sunrise, Grödig, Austria) and concentrations were calculated with Magellan software (Tecan) using the standard curves as allocation base.

### Time-resolved fluorometry immunoassay of saliva and BALF

The concentrations of Hp and CRP in saliva and BALF samples were quantified using previously developed and validated one-step non-competitive sandwich type immunoassays based on time-resolved fluorometry technology [25, 26]. The assays used for Hp and CRP measurements comprise calibration curves with seven standards with concentrations between 10 and 1500 ng/ml approximately. This wide dynamic range allows the quantification of samples with highly varying protein concentrations. For saliva, the optimal sample dilution was 1:10 and 1:2 for Hp and CRP measurements respectively, as reported previously. However, described assays had not been evaluated for BALF samples so far, so that the procedures were modified by using optimised dilutions of 1:100 and 1:10 for Hp and CRP quantifications respectively.

### Bacterial isolation and cultivation

Lung and tonsillar tissue samples, as well as nasal swabs of four control (C1-4) and six infected (I1-6) animals were examined for the presence of *A. pleuropneumoniae*. For bacterial isolation swabs from organ tissue and nostrils were streaked on Columbia sheep blood agar (Oxoid, Wien, Austria). *Staphylococcus aureus* was used as nurse to facilitate the isolation of *A. pleuropneumoniae* from organs carrying a high bacterial background microbiota, such as tonsils and nostrils [1]. Subsequent cultivation of bacteria was performed in PPLO broth (Difco™, Becton, Dickinson and Company, Franklin Lakes, USA) supplemented with 10 mg/l NAD (AppliChem GmbH, Darmstadt, Germany) for molecular analyses or grown as solid cultures supplemented with 14 g/l bacteriological agar (Oxoid) for FTIR spectroscopy (see below). All bacterial cultivations were carried out at 37 °C and 5% CO_2_.

### Serotype 2 specific PCR

Serotype 2 specific PCR using primers for the capsular biosynthesis genes *cps2AB* was performed to confirm the identity of *A. pleuropneumoniae* re-isolated from infected host tissue [27]. Briefly, pelleted bacteria from 2–4 ml liquid culture were re-suspended in 100 μl ddH_2_O and lysed at 100 °C for 10 min. Cell debris was removed by 3 min centrifugation at 13.000 × g. 2 μl supernatant containing 50 ng/μl genomic DNA served as a template for a 25 μl PCR reaction mixture containing 5 μl 5× Green GoTaq® Flexi buffer (Promega, Madison, USA), 2.5 μl 25 mM MgCl_2_, 0.5 μl 20 mM dNTP, 0.25 μl APPcps2F and APPcpsR Primer each (50 pmol), 0.125 μl GoTaq® Flexi DNA Polymerase (5 U/μl; Promega, Madison, USA) and 14.38 μl ddH_2_O. DNA was amplified for 35 cycles with the following parameters: 30 s denaturation at 94 °C, 30 s annealing at 58 °C and 30 s elongation at 72 °C. PCR products were analysed on a 1.5% agarose gel.

### DNA fingerprinting of bacteria

Genetic stability of bacteria was confirmed by M13 - PCR typing of re-isolated *A. pleuropneumoniae* and the original challenge strain grown *in vitro* as previously described by Henderson et al. [28]. Genomic DNA was isolated with the MasterPure™ DNA Purification Kit (Epicentre, Madison, USA) following the manufacturer’s instructions. 2 μl genomic DNA (50 ng/μl) was used as a template for a 25 μl PCR reaction mixture containing 5 μl 5 × Green GoTaq® Flexi buffer (Promega, Madison, USA), 2.5 μl 25 mM MgCl_2_, 0.5 μl 20 mM dNTP, 0.5 μl Primer M13 (50 pmol/μl; 5’-GAGGGTGGCGGCTCT-3’), 0.15 μl GoTaq® Flexi DNA Polymerase (5 U/μl; Promega, Madison, USA) and 14.35 μl ddH_2_O. The amplification was performed using the following parameters: 35 cycles of 35 s of denaturation at 95 °C, 1 min annealing at 40 °C and 2 min of extension at 72 °C. PCR products were analysed on a 2% agarose gel.

### Preparation of crude capsular extract

Crude capsule extracts (CEs) were prepared from *A. pleuropneumoniae* isolates derived from lung and tonsils of the infected pigs by mild water-phenol extraction. In brief, fresh 50 ml liquid cultures of *A. pleuropneumoniae* isolates were grown to an OD_600_ of 0.2 and bacteria were harvested by centrifugation at 6530 × g for 5 min. Per gram of bacterial mass 18 ml 1% phenol were added and capsules were extracted by shaking for 5 min at 37 °C followed by an incubation step at 4 °C for 30 min. Bacterial debris was pelleted by centrifugation at 27000 × g for 30 min at 4 °C. Supernatant was sterile filtrated with a syringe sterile filter (pore size 22 μm) to remove remaining cellular debris. The phenol solution was removed by vacuum centrifugation and CE was re-suspended in ddH_2_O and immediately analysed by FTIR spectroscopy.

### FTIR spectroscopy and spectral data analyses

FTIR spectroscopy was employed to investigate the impact of the host on the metabolism of *A. pleuropneumoniae* re-isolated from organs and tissues of infected pigs. Infrared spectra reflect the biochemical composition of living cells by mirroring the stretching and bending vibrations of proteins, nucleic acids, polysaccharides and fatty acids within different frequency areas [11], which makes FTIR spectroscopy a powerful and highly discriminatory tool for the generation of bacterial metabolic fingerprints and to study host-pathogen interactions [12]. For the generation of a metabolic fingerprint, a loop full of a bacterial mass of the different isolates was cultivated as a lawn on PPLO agar at 37 °C for 24 h and samples were prepared for FTIR spectroscopy as described previously [29, 30]. In brief, one loop-full of bacteria was suspended in 100 μl sterile distilled water. Subsequently, an aliquot of 30 μl cell suspension was transferred to a zinc selenide (ZnSe) optical plate (BrukerOptics GmbH, Ettlingen, Germany) and dried for 40 min at 40 °C. Infrared absorption spectra were recorded in transmission mode in the range of 4000 to 500 cm^−1^, with a resolution of 6 cm^−1^, zero-filling factor 4 and Blackmann-Harris 3-term apodization by the aid of a HTS-XT microplate adapter coupled to a Tensor 27 FTIR spectrometer (BrukerOptics GmbH). An average spectrum of 32 scanned interferograms was calculated with background subtraction for each spectrum. Spectral data were processed and analysed and subsequent chemometric analysis was performed using OPUS software (Version 7.2; BrukerOptics GmbH). The frequency range 1150–1100 cm^−1^ in the polysaccharide region (1200–900 cm^−1^) provided the maximum discriminatory information to assess the tissue-related bacterial metabolic fingerprints by hierarchical cluster analysis (HCA). Most characteristic frequency areas to assess differences specifically related to the bacterial capsule were the polysaccharide region (1200–900 cm^−1^) and the protein region (1800–1500 cm^−1^) [11]. Dendrograms of 2^nd^-derivative spectra (9-point Savitzky-Golay filter) were generated using the Ward’s algorithm with normalisation to repro level 30. Measurements of CE were performed with minor alterations as previously described [31].

### Statistical analysis

Statistical data analysis was performed for protein and mRNA level and for the clinical score by using the SPSS software (2011, IBM, SPSS Statistics for Windows, Version 20.0, Armonk, NY, IBM Corp.). Since data were not normally distributed, non-parametrical tests were performed. For statistical evaluation of group differences between infected and sham control pigs the Wilcoxon rank sum test was used. Parameters in paired samples prior to and after infection within one group were compared by the Wilcoxon Signed-Rank test.

## Results

### Clinical and post-mortem findings

**Fig. 1:**
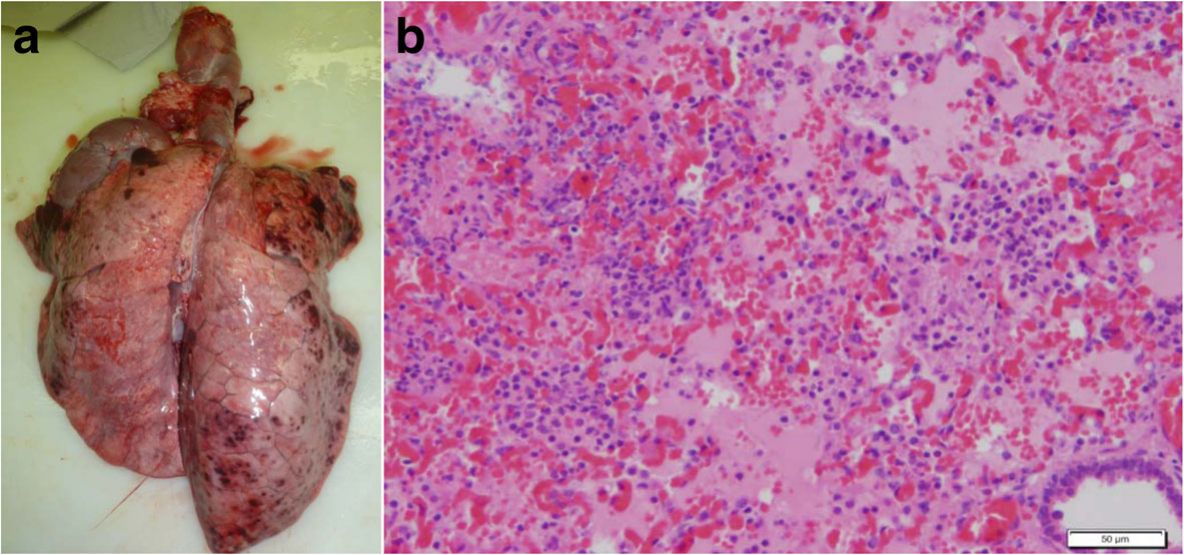
Pathological lung tissue alterations in a pig 8 hpi with *A. pleuropneumoniae* serotype 2. **a** Macroscopic lung alterations are characterised by multifocal and disseminated haemorrhagic lung tissue consolidations; **b** Histopathological findings are dominated by fibrinous exudates in alveolar spaces and interlobular septa (H&E stain; bar = 50 μm)

Four hours after infection all pigs experimentally infected with *A. pleuropneumoniae* showed signs of respiratory disease such as dyspnoea, open mouth breathing and coughing. Rare episodes of vomiting were observed in both, infected and control animals, most likely due to a side effect of anaesthesia. Control animals did not display any sign of respiratory disease (Table 1). Successful experimental infection was additionally confirmed by macroscopic and microscopic pathological lung alterations. In the lungs of all infected animals red foci of consolidation and multifocal haemorrhagic lesions associated with interlobular oedema were found (Fig. 1a). In one animal scattered layers of fibrin on the *Pleura visceralis* indicated an incipient pleurisy. Lung lesion scores of infected animals were significantly higher than those of controls as shown in Table 1 (*p* ≤ 0.01). Microscopic lung tissue alterations in infected pigs were dominated by a severe neutrophilic infiltration as well as fibrin exudation into the alveolar spaces and interalveolar septa leading to an obstruction of bronchioli (Fig. 1b). The histological diagnosis was a fibrinopurulent, necrotizing and haemorrhagic pleuropneumonia (Table 1). Control pigs showed no histological signs of pleuropneumonia.

**Table 1:** Clinical score (CS), lung lesion score (LLS) and histological evaluation of lung lesions in control and infected animals

Group	Clinical score	Lung lesion score	Histological evaluation
Control (*n* = 4)	0 (0–0.75)	0 (0–0.6)	Moderate infiltrates of neutrophils and macrophages in interalveolar septa
Infected (*n* = 6)	**4** (2.75–5.5)**	**7.39** (3.1–16.56)**	Fibrinopurulent, necrotizing, hemorrhagic pleuropneumonia

**p ≤0.01; Wilcoxon rank sum test

CS prior to death (8 hpi) and LLS at the time of the necropsy are expressed as median (interquartile range). Significant values are marked in bold

### Cytological findings in blood

**Fig. 2:**
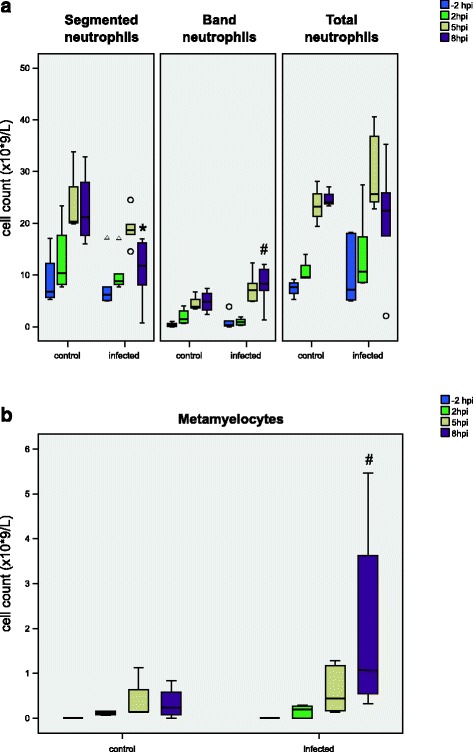
Cytological findings in the blood during the course of infection. The absolute numbers of mature and immature neutrophils (**a**: total, segmented, band neutrophils, **b**: metamyelocytes) prior to and at 2, 5 and 8 hpi of control (*n* = 4) and infected (*n* = 6) animals are shown. Box-plots show median (line within the box), 25^th^ and the 75^th^ percentiles (box), 1.5 times the interquartile range (whiskers) and outliers (circles and triangles). *p ≤ 0.05 Wilcoxon rank sum test in comparison to control, # *p* ≤ 0.05 Wilcoxon signed-rank test in comparison to sampling prior to infection within the same group

At no time point after infection did the total number of neutrophils in the blood differ between the two groups. Nevertheless, differences were detectable when considering singularly granulocyte fractions. As shown in Fig. 2, eight hours after infection segmented neutrophils in infected animals decreased to a significantly lower level than in control pigs, while a significant increase in immature granulocytes, both band cells and metamyelocytes, was observed in infected pigs in comparison to the sampling prior to infection. Already five hours after infection band neutrophils rose in infected pigs (Fig. 2).

BALF was harvested eight to ten hours after infection from the isolated lungs. Significantly (*p* ≤ 0.01) higher total leukocyte cell counts, as well as a trend (*p* = 0.057) in lymphocytes and alveolar macrophages, were observed in infected pigs (Table 2).

**Table 2:** Concentrations of cytokines (IL-6, IL-1 and TNF-α), acute phase proteins (CRP, Haptoglobin) and cells in different bodily fluids from control and infected animals

	Control (*n* = 4)^a^	Infected (*n* = 6, ^c^ *n* = 3)
BALF Cytokines:		
IL-6 (pg/ml)	ND	434.2 (ND-2491.5)
IL-1β (pg/ml)	ND	1166.8 (ND-2808.1)
TNF-α (pg/ml)	129.5 (ND-410.4)	ND
BALF acute phase proteins:		
CRP (ng/ml)	89.7 (39.5–189.6)	389.7 (192.6–554.4)
Hp (μg/ml)	6.7 (5.2–9.1)	6.2 (5.1–7.5)
BALF Cells:		
Leukocytes (×10^9^/l)	2.9 (1.8–5.1)	**11.4** (7.8–16.9)**
Lymphocytes (×10^9^/l)	0.1 (0–0.3)	^c^2.5 (0.8–6.1)
Alveolar Macrophage (×10^9^/l)	1.7 (1.5–2.3)	^c^6.2 (4.5–10.6)
	Infected (pre-infection)^b^	Infected (8 hpi)
Serum acute phase proteins:		
CRP (μg/ml)	49.7 (18.4–71.5)	**202.2** (93.9–309)*
Hp (mg/ml)	0.7 (0.2–1.1)	0.7 (0.5–1.3)
Saliva acute phase proteins:		
CRP (ng/ml)	3 (1.3–7.4)	27.9 (8.4–37.6)
Hp (μg/ml)	0.4 (0.2–1)	**1.9** (1.3–3.6)*

Results are expressed as median values (interquartile range). ^a^Differences between control and infected group in the BALF immediately after death. *p ≤ 0.05

**p ≤ 0.01 Wilcoxon rank test. ^b^Differences within the infected group between the last time point of sampling (8 hpi) and prior to infection. *p ≤ 0.05 Wilcoxon signed ranks test. ^c^Lymphocytes and alveolar macrophages were not detectable in the BALF of three animals out of the infection group

ND = not detectable because below detection limit

Significant values are marked in bold

### Gene expression in host tissue

**Fig. 3:**
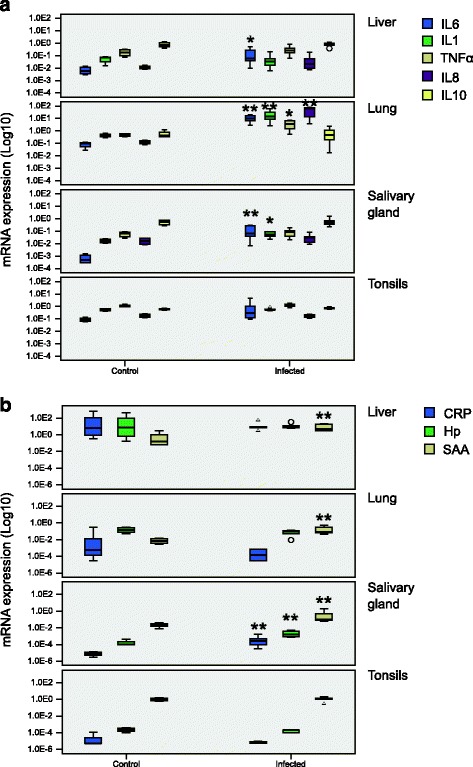
Cytokine and acute phase protein mRNA analyses. mRNA expression of (**a**) cytokines IL-6, IL-1, TNF-α, IL-8, IL-10 and (**b**) acute phase proteins CRP, C-Reactive Protein; Hp, Haptoglobin and SAA, Serum Amyloid A in different tissues of control (*n* = 4) and infected (*n* = 6) animals. Box-plots show median (line within the box), 25^th^ and the 75^th^ percentiles (box), 1.5 times the interquartile range (whiskers) and outliers (circles and triangles). The results were normalised to reference genes (Actin and Cyclophilin A) and to the internal standard. *p ≤ 0.05, **p ≤ 0.01

Gene expression of inflammatory cytokines (IL-6, IL-1 and TNF-α), chemokine IL-8, and the anti-inflammatory cytokine IL-10 was evaluated in liver, lung, salivary gland and tonsils (Fig. 3). Gene expression of iNOS, HO1, IL-2, IL-4 and IFN-γ was below the detection limit and for this reason is not mentioned further in this study (data not shown). In infected animals, IL-6 expression was significantly up-regulated in the liver (*p* ≤ 0.05), lung and salivary gland (both *p* ≤ 0.01) in comparison to control pigs, but in the tonsils, only a tendency was detected (*p* = 0.06). In the lung tissue of infected animals, all pro-inflammatory cytokines and IL-8 were significantly (*p* ≤ 0.05) up-regulated. In addition, IL-1 expression was significantly increased (*p* ≤ 0.05) in the salivary gland of infected pigs. No significant differences were found for IL-10 expression in any tissue. In the tonsils none of the investigated parameters were changed (Fig. 3A). The mRNA expression of acute phase proteins (CRP, Hp and SAA) was also assessed in the same tissues (Fig. 3B). Serum amyloid A (SAA) expression was found to be significantly up-regulated (*p* ≤ 0.01) in all tissues apart from tonsils. In the salivary gland, the expression of all acute phase proteins was significantly up-regulated (*p* ≤ 0.01), while in the tonsils no difference in gene expression in comparison to the control group was found.

### Protein expression in serum, BALF and saliva

Concentrations of pro-inflammatory cytokines (IL-6, IL-1, TNF-α) were determined in serum and BALF and acute phase proteins (CRP and Hp) were quantified in serum, BALF and saliva. In sera of infected animals, no significant changes in inflammatory cytokines were detected (data not shown).

Interestingly, TNF-α was not detected in BALF after infection. In contrast, the median concentrations of IL-6 and IL-1 were increased in infected animals while remaining below the detection limit in control pigs. Acute phase proteins behaved similarly in serum and BALF. While Hp concentrations remained unchanged throughout the study, CRP increased significantly at eight hours after infection (Table 2.).

The concentrations of CRP and Hp analysed in saliva showed increased levels in the infected animals. Specifically, at the last time point, Hp increased significantly (*p* ≤ 0.05) whereas CRP showed only a trend (*p* = 0.07). In control animals no changes were detected.

### Metabolic fingerprinting of *A. pleuropneumoniae* re-isolated from host tissues

**Fig. 4:**
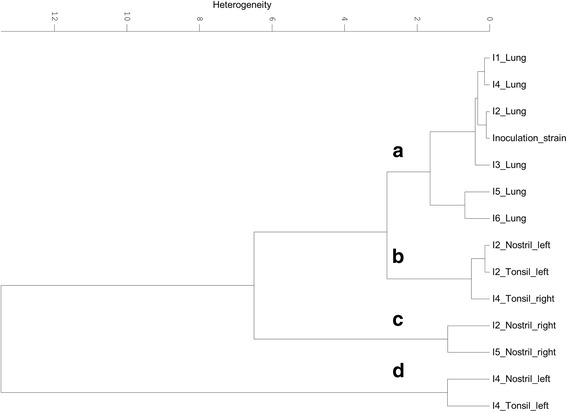
Hierarchical cluster analysis of FTIR spectra recorded from *A. pleuropneumoniae* serotype 2 re-isolated from different organs of infected pigs. Isolates from different organs of the infected pigs 1–6 (I1-I6) and the strain used for infection were grown for 24 h on PPLO at 37 °C and 5% CO_2_, subsequently subjected to FTIR spectroscopy and hierarchical cluster analysis was performed. For calculation of the dendrogram the FTIR spectral regions of 1150 to 1100 cm^−1^ and the Ward’s algorithm were used *A. pleuropneumoniae* re-isolated from the lung of all six animals (I1-6) group and the inoculation strain group together in cluster (**a**), while the bacteria isolated from the tonsils and the nostrils group in cluster (**b**–**d**)

*A. pleuropneumoniae* was isolated from the lung of all six, the tonsils of two and the nostrils of three infected animals (Table 3). Serotype 2 specific PCR (APPcps2 PCR) was used to confirm that the isolated *A. pleuropneumoniae* colonies are indeed derived from the *A. pleuropneumoniae* serotype 2 strain used for infection, [27]. The clonality and genetic stability of the isolates were checked by DNA fingerprinting via M13-PCR [28]. All isolates showed the same band pattern via M13-PCR indicating that all isolates are indeed progenitors of the strain used for infection (data not shown). FTIR spectra of all *A. pleuropneumoniae* isolates from the host tissues were recorded and subjected to chemometric analysis. Hierarchical cluster analysis (HCA) of the normalised and pre-processed spectral data revealed a distinct organ-specific clustering of the isolates, indicating organ-specific impacts on the metabolism of host passaged *A. pleuropneumoniae*. From the recorded IR spectra, the spectral window of 1150 to 1100 cm^−1^, representing a part of the polysaccharide region, showed the highest discriminatory power, resulting in 4 major clusters (Fig. 4). While cluster A covers all isolates from the lung and the inoculation strain, isolates of the upper respiratory tract (tonsillar and nostril isolates) cluster apart from cluster A in the three clusters B-D (Fig. 4). Further passaging showed that the bacterial metabolic adaptation remained stable for the first passages. Consecutive cultivation under laboratory conditions revealed that these metabolic changes are reversible. HCA of FTIR spectral data from bacteria passed 5 times under laboratory conditions showed that isolates from the upper and lower respiratory tract are clustering together (see Additional file 2). The latter results indicate that the observed adaptation is indeed a metabolic adaptation triggered by the organ-specific environment, which is lost over time due to consecutive cultivation under laboratory conditions.

**Table 3:** *A. pleuropneumoniae* re-isolated from different tissues of acutely infected animals

		Animal					
Tissue		I1	I2	I3	I4	I5	I6
Lung		x	x	x	x	x	x
Nostril	Left	-	x	-	x	-	-
	Right	-	x	-	-	x	-
Tonsil	Left	-	x	-	x	-	-
	Right	-	-	-	x	-	-

### Analysis of *A. pleuropneumoniae* CEs

**Fig. 5:**
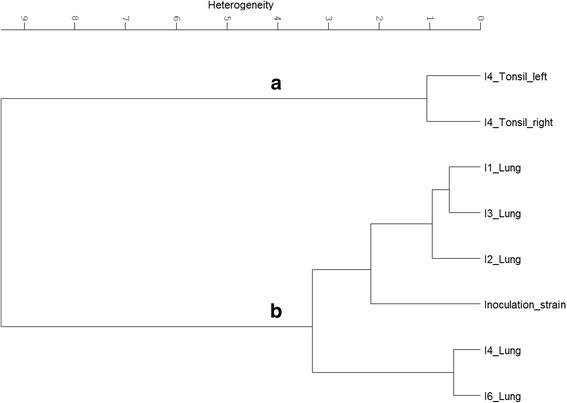
Hierarchical cluster analyses of FTIR spectroscopic data from CEs of the *A. pleuropneumoniae* serotype 2. Hierarchical cluster analysis was performed using recorded FTIR spectra from the CEs of the infection strain grown *in vitro* and after re-isolation from different organs of the infected pigs 1–4 and 6 (I1-4 and I6): isolates from the lung of I1-4 and 6 as well as the inoculation strain (Cluster **b**), isolates from the left and right tonsil of I4 (Cluster **a**). For calculation of the dendrogram the Ward’s algorithm and the FTIR spectral regions of 1200–900 cm^−1^ and 1800–1500 cm^−1^ were used

**Fig. 6:**
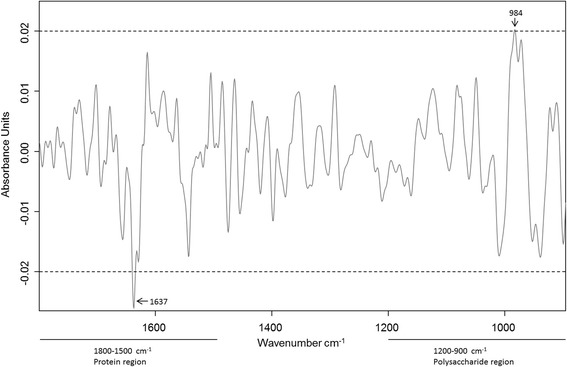
Differential FTIR spectral analyses. The second derivate, vector-normalised average spectra were calculated from CE of re-isolated *A. pleuropneumoniae* Serotype 2 from the left and right tonsil of the infected pig 4 (I4) and the lung of the infected pigs 1–4 und 6 (I1-4 and I6), respectively. The average spectrum of the lung of I1-4 and 6 was subtracted from the average spectrum of the tonsils of I4. Most pronounced differences could be detected in the frequency area of 1800–900 cm^−1^ and can be related to the protein region (1800–1500 cm^−1^) and the carbohydrate region (1200–900 cm^−1^)

The metabolic fingerprints of the *A. pleuropneumoniae* lung and upper respiratory tract (nostrils and tonsils) isolates obtained by FTIR spectroscopy showed remarkable differences in the polysaccharide and the protein region. Since the capsule, which is an important virulence factor of *A. pleuropneumoniae* [32], mainly consists of polysaccharides, CE from the re-isolated bacteria were prepared. In order to retain CE associated proteins, a mild aqueous phenol capsule extraction method without further purification was used for the latter. The extracted CE was subjected to FTIR spectroscopy and subsequent chemometric analysis. HCA of the FTIR spectral date of the CEs, using the frequency areas 1800–1500 cm^−1^ (protein region) and 1200–900 cm^−1^ (polysaccharide region), revealed two major clusters: one cluster comprising of the CE spectra from the strain used for infection and its isolates derived from the lung of the infected pigs I1-4 and I6, and one cluster comprising the CE spectra from the tonsil isolates of the infected pig I4 (Fig. 5). Differential FTIR spectral analysis was carried out to search for host site-specific imprints in the CE from the re-isolated bacteria. Therefore, subtraction of a second derivate, vector-normalised, average FTIR spectrum of the CE from *A. pleuropneumoniae* lung isolates from the average spectrum of tonsillar isolates was performed (Fig. 6). The comparison between the CE of isolates revealed significant alterations within the protein (1800–1500 cm^−1^) and carbohydrate (1200–900 cm^−1^) regions. Higher amounts of substances absorbing at 1637 cm^−1^ were recorded in CE of lung isolates. This band can be assigned to amide I of β-pleated sheet structures [33]. Another significant difference could be detected in the tonsillar CE at 984 cm^−1^ compared to the CE of the lung. Being part of the carbohydrate region this band falls into the spectral range in which O–C, C–O structures dominated by ring vibrations of carbohydrates C–O–P and P–O–P absorb [34].

## Discussion

Early induced immune response is the first line of defence against many common microorganisms, including *A. pleuropneumoniae*. Its failure in eliminating the pathogen can result in the development of a chronic and persistent infection status [2, 35]; for this reason, the acute stage of porcine pleuropneumonia, where early immune response usually takes place, was investigated in this study. To induce an acute stage of infection an intratracheal infection route was applied. The experimental model used in this study resulted in diffuse bilateral pneumonia in all infected pigs and the LLS mirrored clinical signs, thus confirming the reproducibility of infection. These results are in line with previous studies using other *A. pleuropneumoniae* serotypes for experimental infection [16]. The intratracheal infection model used ensures equally distributed lesions and partially overcomes the limitation of the small sample size in this study by a reduction in the variability commonly associated with other routes of infection [16].

After eight hours of infection, the drop in segmented neutrophils in combination with a rise in band cells and metamyelocytes reveals the kinetics of the cellular innate immune response associated with a fast migration of immature band cells from the bone marrow to the blood as well as a migration of mature neutrophils from the blood stream to the site of infection. Rapid neutrophilic infiltration and high levels of IL-8 expression in lung tissue of infected pigs confirm the key role of this neutrophil-chemokine interaction in the pathogenesis of the disease [8, 16].

In serum, no changes in IL-1 concentrations and only slight increases in IL-6 and TNF-α were recorded within the first eight hours after infection, which is in accordance with results of previous studies [8, 36]. Fossum et al. (1998) reported an increase in IL-6, as the earliest detectable pro-inflammatory cytokine, at 20 hpi with *A. pleuropneumoniae* serotype 2 [36]. Recently, a study from Wyns et al. (2015) showed that all three cytokines increased after the experimental infection and the mean peak concentrations of TNF-α and IL-6 in serum were registered at 12 and 10 hpi respectively [37]. In contrast to these findings for pro-inflammatory cytokines, serum CRP concentration raised eight hours after infection. Serum Hp concentration was not affected during the infection trial.

Our findings of a 4-fold increase in serum CRP is in line with the previously described range of a 2- to 9-fold increase after infection. The normal CRP concentrations in the plasma of healthy pigs vary from 11–77 μg/ml with a high individual variation [38, 39], which is also in accordance with our findings. In contrast to CRP, the slow reacting acute phase protein Hp remained at a physiological level (0.19–0.52 mg/ml) [5, 40] in the serum from both groups. In contrast to serum data, IL-6 and IL-1 were increased in BALF from infected animals; leading to the idea that acute bacterial respiratory tract infection might induce localised rather than systemic cytokine responses. However, TNF-α protein was not detectable in infected pigs, even though its mRNA was found to be highly expressed in the lung. The same outcome was observed in a former study [8]. A study in mice showed the ability of surfactant protein A to enhance the production of secretory leukoprotease inhibitor (SLPI), which is known to induce down-regulation of TNF-α production by inhibiting LPS-induced NF-kB activation [41]. Thus, it is tempting to speculate that LPS-induced NF-kB activation might also be involved in the down-regulation of TNF-α detected in BALF of infected pigs compared to control. Nevertheless, further studies are necessary to test the latter hypothesis.

Although the Waldeyer’s ring was bypassed by the intratracheal inoculation route the examined innate immune responses were not confined to the lung, but rather disseminated to organs of the upper respiratory tract. While pro-inflammatory cytokines were primarily induced in the lung tissue of all infected animals, the salivary gland was the main site of expression of acute phase proteins, IL-6 and IL-1. The latter finding was unexpected, because it is in contrast to the lack of immune reaction in tonsillar tissue, although both organs might have had pathogen contact at the same time but later than the lung.

Despite their common localisation, the salivary gland and tonsils showed a completely different pattern in the host immune response. Neither acute phase protein nor pro-inflammatory cytokine mRNAs were differentially expressed in tonsillar tissue. Acute phase protein extra hepatic production in peripheral lymphoid tissue was previously described during acute *A. pleuropneumoniae* infection [5]. However, the impact of the salivary gland in extra hepatic production of acute phase proteins is largely unknown. Immuno-histochemical findings by Gutierrez et al. (2012) revealed localisation of Hp in the glandular acini and duct epithelial cells of the salivary gland [6]. In our study, not only Hp and SAA, but also CRP was significantly increased in pigs infected with *A. pleuropneumoniae*. This expression was evident both, at a transcriptional level and at the level of the protein, since Hp and CRP reached higher values also in saliva from infected animals. Whether this prominent implication of the salivary gland during acute bacterial infection is linked to the direct presence of *A. pleuropneumoniae* in this site is hitherto unknown. Nonetheless, the detection of the bacterium in the tonsils sited in anatomical proximity, and the reported susceptibility of the salivary gland to *A. pleuropneumoniae* colonisation [42], is a hint that the bacteria could invade this organ. Interestingly, bacteria could be re-isolated from tonsillar tissue, as a consequence of coughing or transport by the mucocillary escalator. However, a host immune response in tonsillar tissue, which would have been indicated by an increase in cytokine or acute phase protein expression, was not detected (Fig. 3).

During the last two decades, FTIR spectroscopy has become a well-established technique for identification of microorganism on the species and subspecies level [11]. Due to its high discriminatory power, FTIR spectroscopy is increasingly employed not only for bacterial identification but also for studying environmental impacts, such as abiotic stress or host genotypes, on the metabolic fingerprints of bacteria [12, 43]. Thus, FTIR spectroscopy represents a suitable tool for exploring the host impacts on *A. pleuropneumoniae* in parallel to the analyses of host reactions in response to the bacterial infection.

In the first step, *A. pleuropneumoniae* re-isolated from different host tissues was subjected to molecular analysis to confirm its genotypic identity with the strain used for inoculation. All isolates were positive in the *A. pleuropneumoniae* serotype 2 specific PCR and showed the same M13-PCR profiles. In contrast to the results from the molecular typing, FTIR spectral analysis revealed a distinct clustering of the isolates. The isolates clustered according to the tissue of re-isolation (Fig 4), reflecting the physiological metabolic adaptation due to their movement from the lower to the upper respiratory tract (Fig. 4–5). This might be indicative for different adaptation strategies, depending on the body compartment of colonisation or infection. Notably, the inoculation strain forms one cluster with the lung isolates while the isolates from the upper respiratory tract formed a separate cluster. This observation fosters out hypothesis that the bacteria are indeed rapidly adapting to the upper respiratory tract, which is reflected in their metabolic fingerprints that are distinct from the ones of the lung isolates and the inoculation strain. Furthermore, our results revealed that the laboratory culture medium (PPLO) and culture conditions used for growth of the inoculation strain mimick the host environment that bacteria are facing during acute infections in the lung (Fig. 4). As shown previously, growth conditions can significantly impact the metabolism and the expression of virulence factors of bacterial pathogens [12] and must be considered when inoculation samples for infection studies are prepared. The observed phenotypical metabolic adaptation of *A. pleuropneumoniae* was reversible, which is in line with recent findings on the loss of host environmentally triggered memory effects over time reported from other bacteria [12, 44, 45]. Since isolation of *A. pleuropneumoniae* from tonsils and nostrils of swine not showing any clinical signs is common, the pathogen is considered to persist at these sites of infection [46]. Thus, when colonizing various host organs, differences in metabolic adaptations may have facilitated a persistence of *A. pleuropneumoniae*. Indeed, the HCA revealed a unique cluster for the lung isolates, while the isolates of the upper respiratory tract were more diverse (Fig. 4). The diversity observed among the latter isolates might reflect the individual appearance of acute disease symptoms, such as coughing.

The most pronounced influence on the metabolic fingerprint during bacterial host organ-specific adaptation was attributed to the spectral range of 1150–1100 cm^−1^. This frequency area is part of the polysaccharide region (1200–800 cm^−1^), which is dominated by a complex sequence of peaks associated to stretching ring vibrations of carbohydrates (C─O─C, C─O─P) [47]. Since a comparison of FTIR spectra of the CEs from *A. pleuropneumoniae* from the tonsils with the FTIR spectra recorded from the lung isolates revealed significant differences within their protein and carbohydrate compositions (Fig. 6), it is tempting to speculate that the capsule of the bacteria has an influence on the specific metabolic adaptation of the bacterium to the different host organs. The stronger appearance of β-sheet structures in the CEs from lung isolates indicates a shift towards a higher expression of β-sheet carrying proteins. To decipher the bacterial molecules and mechanisms involved in observed host tissue specific adaptation of *A. pleuropneumoniae* detailed quantitative and qualitative carbohydrate and protein analyses of the capsule are needed, which are clearly beyond the scope of our current work. Preliminary results from an ongoing study also point towards a reduced production of capsule material in the tonsil isolates (Frömbling et al., unpublished) and for *S. aureus* it has already been reported that the loss of capsule expression is advantageous to establish and maintain a chronic infection in humans as well as in animals (e.g. chronic osteomyelitis, bovine mastitis) [48]. Based on our results, it is tempting to speculate that the capsule formation is important for bacterial adaptation to the porcine tonsils as one step towards deciphering persistence of *A. pleuropneumoniae* in infected pig herds.

## Conclusions

Altogether, our results indicate an important role of the salivary gland in oral immunity, already eight hours after infection. Contrarily to the lung, *A. pleuropneumoniae* was not able to provoke any immune response in the tonsils. Thus, the specific changes observed in the metabolic fingerprints of *A. pleuropneumoniae* are presumably crucial for bacterial adaptation to porcine tonsils. Further studies will be necessary to decipher the exact role and contribution of the tonsils as a reservoir of host adapted *A. pleuropneumoniae* for the development and establishment of chronic infections.

## Additional files

Additional file 1: Information about IL8 primers and optimised qPCR assays. More details about the optimisation and validation of qPCR assays for target gene-specific primers in the pig are included. Particularly in the figure is shown that the suitability of the newly designed primers was verified in separate experiments by performing of a cDNA pool. In melt curve and amplification plots samples are shown in green while controls (no reverse transcription control (NRT) and no template control (NTC)) are shown in yellow and orange respectively. Additionally, an agarose gel electrophoresis of the PCR products of undiluted cDNA pool and controls was performed. (DOCX 349 kb)
Additional file 2: Hierarchical cluster analysis of FTIR spectroscopic data recorded from A. pleuropneumoniae serotype 2 after 2 and 5 passages under laboratory conditions. After re-isolation from different organs of the infected pigs 1–6 (I1-I6) recorded FTIR spectroscopic data of 2 and 5 passages on laboratory medium were subjected to hierarchical cluster analyses. After two passages nostril and tonsil isolates (upper respiratory tract isolates) cluster apart from all lung isolates, while passaging for five times leads to two intermingled clusters of lung and upper respiratory tract isolates as well as to a decrease in heterogeneity between the samples. For calculation of the dendrogram, the FTIR spectral regions of 900 to 1200 cm^−1^ and 1500 to 1800 cm^−1^ and Ward’s algorithm were used. (PPTX 63 kb)
