# Supplementary material for: Host-pathogen interplay at primary infection sites in pigs challenged with Actinobacillus pleuropneumoniae
Source: BMC Vet Res. 2017 Feb 28;13:64. doi: 10.1186/s12917-017-0979-6 (PMC5329957; doi:10.1186/s12917-017-0979-6)
Supplement: Additional file 1: — Information about IL8 primers and optimised qPCR assays. More details about the optimisation and validation of qPCR assays for target gene-specific primers in the pig are included. Particularly in the figure is shown that the suitability of the newly designed primers was verified in separate experiments by performing of a cDNA pool. In melt curve and amplification plots samples are shown in green while controls (no reverse transcription control (NRT) and no template control (NTC)) are shown in yellow and orange respectively. Additionally, an agarose gel electrophoresis of the PCR products of undiluted cDNA pool and controls was performed. (DOCX 349 kb) [file 12917_2017_979_MOESM1_ESM.docx]

**Additional file 1: Optimisation and validation of qPCR assays for target gene-specific primers in the pig.**

**Table :** Information about Intron-spanning primers.

| **Target** | **Accession number** | **Sequence** | **Position. on plus-strand** | **Product-length (bp)** | **Exon junctions in** | **Intron size**  **(bp)** |
| --- | --- | --- | --- | --- | --- | --- |
| **IL8** | NM_213867.1 | AACAGCCCGTGTCAACATGA | 68 | 125 | product | ~1000 |
|  |  | TGCACTGGCATCGAAGTTCT | 192 |  |  |  |

**Table:** Optimised protocol and validation studies using cDNA pool dilution series.

| **Target** | **Annealing**  **temp (°C)/time (sec)** | **Extension**  **temp (°C)/time (sec)** | **ΔCt**  **(RT+ to RT-)** | **slope** | **Correlation Coefficient (Pearson) R^2^** | **Verified dynamic range** |
| --- | --- | --- | --- | --- | --- | --- |
| **IL8** | 62/30 | 72/40 | n.d. | -3.545 | 0.998 | 10^6^ |


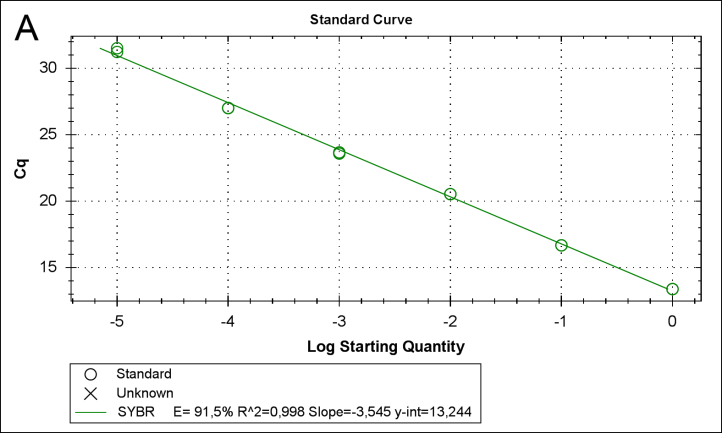

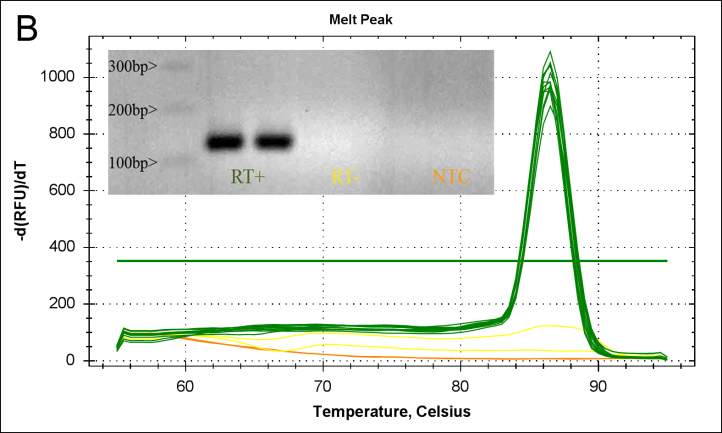

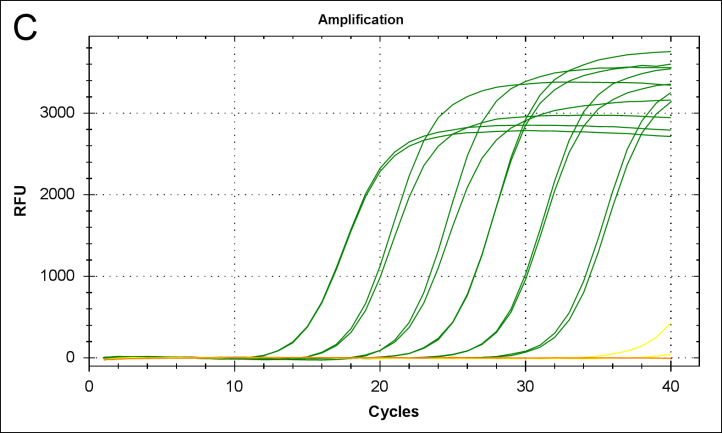


**Figure** The suitability of the newly designed primers was verified in separate experiments by performing of a cDNA pool (**A**). In melt curve (**B**) and amplification plots (**C**) samples are shown in green while controls (no reverse transcription control (NRT) and no template control (NTC)) are shown in yellow and orange respectively. In the inlet in B shows an agarose gel electrophoresis of the PCR products of undiluted cDNA pool and controls.
