# Supplementary material for: Host-pathogen interplay at primary infection sites in pigs challenged with Actinobacillus pleuropneumoniae
Source: BMC Vet Res. 2017 Feb 28;13:64. doi: 10.1186/s12917-017-0979-6 (PMC5329957; doi:10.1186/s12917-017-0979-6)
Supplement: Additional file 2: — Hierarchical cluster analysis of FTIR spectroscopic data recorded from A. pleuropneumoniae serotype 2 after 2 and 5 passages under laboratory conditions. After re-isolation from different organs of the infected pigs 1–6 (I1-I6) recorded FTIR spectroscopic data of 2 and 5 passages on laboratory medium were subjected to hierarchical cluster analyses. After two passages nostril and tonsil isolates (upper respiratory tract isolates) cluster apart from all lung isolates, while passaging for five times leads to two intermingled clusters of lung and upper respiratory tract isolates as well as to a decrease in heterogeneity between the samples. For calculation of the dendrogram, the FTIR spectral regions of 900 to 1200 cm−1 and 1500 to 1800 cm−1 and Ward’s algorithm were used. (PPTX 63 kb) [file 12917_2017_979_MOESM2_ESM.pptx]

## Slide 1
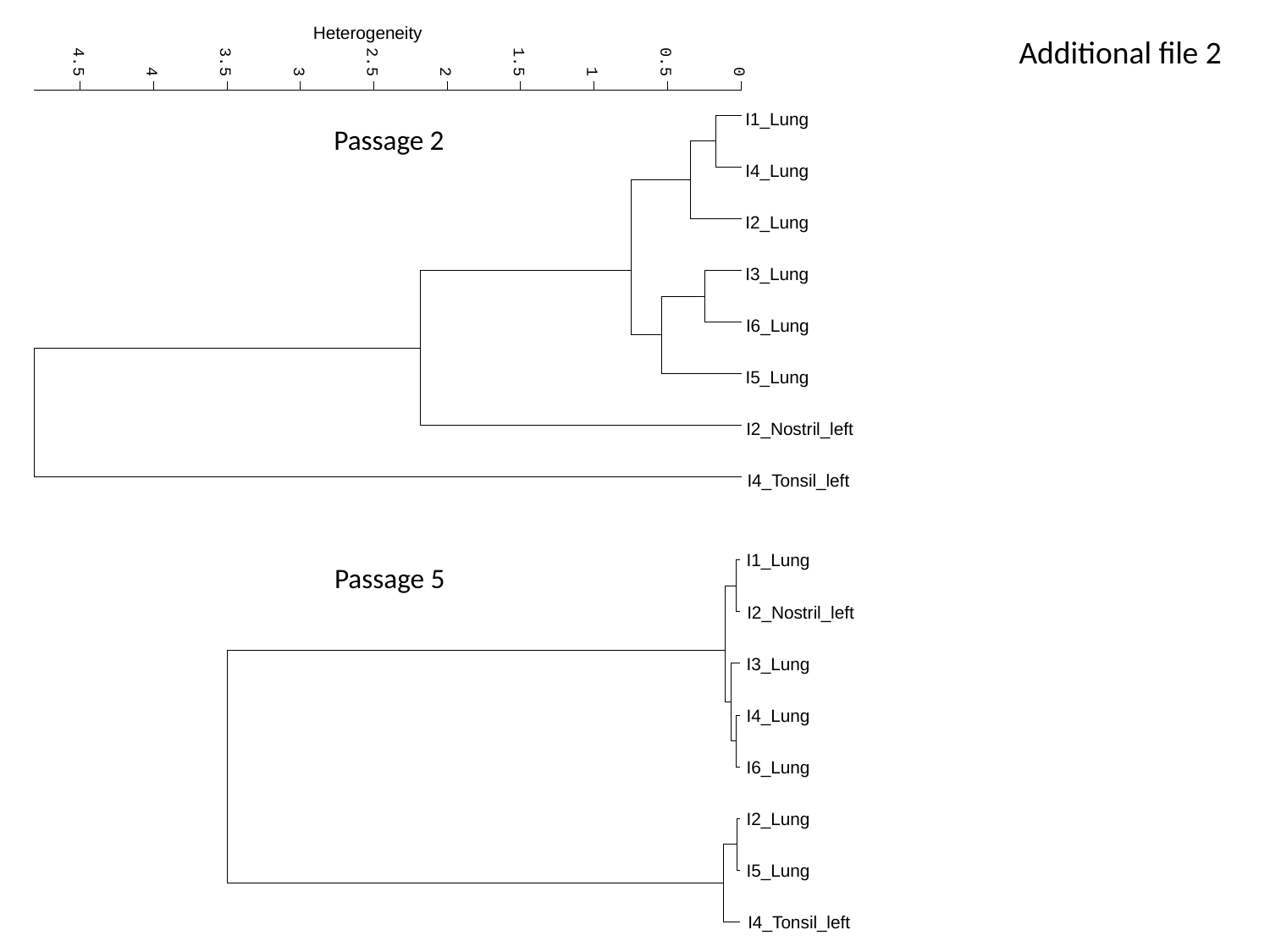

Heterogeneity
4.5
 4
3.5
 3
2.5
 2
1.5
 1
0.5
 0
I1_Lung
I4_Lung
I2_Lung
I3_Lung
I6_Lung
I5_Lung
I2_Nostril_left
I4_Tonsil_left
I1_Lung
I2_Nostril_left
I3_Lung
I4_Lung
I6_Lung
I2_Lung
I5_Lung
I4_Tonsil_left
Additional file 2
Passage 2
Passage 5
